# In Situ Spinel Formation in a Smart *Nano*-Structured Matrix for No-Cement Refractory Castables

**DOI:** 10.3390/ma13061403

**Published:** 2020-03-19

**Authors:** Dominika Madej, Karina Tyrała

**Affiliations:** Department of Ceramics and Refractories, Faculty of Materials Science and Ceramics, AGH University of Science and Technology, al. A. Mickiewicza 30, 30-059 Krakow, Poland; ktyrala@agh.edu.pl

**Keywords:** refractories, alumina-spinel castables, hydrotalcite, HT-XRD, ^27^Al MAS-NMR, nano-structured materials

## Abstract

The hydration of an equimolar mixture of MgO and Al_2_O_3_ nano-powders has been proven to be an effective way to synthesize Mg_6_Al_2_CO_3_(OH)_16_∙4H_2_O as a component of a nano-structured matrix and magnesia-alumina spinel precursor for high-performance cement-free corundum-spinel refractory castables. (Mg_3_)–OH–brucite sites (417 °C) formed initially within the magnesia–alumina hydrating blended paste were replaced with (Mg_2_Al)–OH and (Mg_3_)–OH hydrotalcite sites, which were dehydroxylated at 420 °C and 322 °C, respectively. This reorganization was connected with the incorporation of anions and water molecules in the interlayer spacing of hydrotalcite, which was dehydrated at 234 °C. Hence, the thermal decomposition of a nano-structured matrix system containing mainly Mg_6_Al_2_CO_3_(OH)_16_∙4H_2_O consists of a complex sequence of dehydration, dehydroxylation and decarbonization, and this finally leads to the formation of inverse spinel MgAl_2_O_4_ and periclase MgO through many intermediate stages containing the mixed tetrahedral-octahedral Al phase and MgO-like structure. Hence, the hydraulic bond that primarily existed was replaced by a ceramic bond at a relatively low temperature, i.e., 700 °C, where a spinel was formed. Important changes in oxygen coordination polyhedra around Al^3+^ in the dehydrated-dehydroxylated hydrotalcite occurred between 600 and 1100 °C.

## 1. Introduction

Cement-free castables (no-cement-content (NCC) castables) have been dynamically developed in recent years because of many reasons: the amount of water required to affect chemical bonding is lower in comparison to the conventional castables; the resulting effective dewatering of castables and both the removal of physically absorbed and chemically bonded water; the absence of lime favorably improves the high temperature properties of castables but they also exhibit outstanding chemical, physical and mechanical properties [1]. The binding systems play a relevant role in workability, dry-out, green mechanical strength, both mechanical and thermomechanical properties and also corrosion resistance. The state-of-the-art nano-scaled alternative binding systems to calcium aluminate cement (CAC) binding have been extensively developed in recent years [2]. The most recent achievements in this field include coagulating binders such as colloidal silica or alumina [1,3], ultrafine-SiO_2_ and MgO [4,5,6], ultrafine-Al_2_O_3_ and MgO [7,8,9,10] and a suspended colloidal precursor of mullite and spinel [3]. The recent findings from the literature [11] have clearly shown that, e.g., the nanoscale alumina reinforced alumina-spinel matrix castables exhibit a significant improvement in the thermal shock resistance or the mechanical strength of colloidal binder containing castables increases due to higher sinterability of colloidal particles as the temperature increases [12]. Using nano-scaled particles instead of micro-scaled particles as magnesia and alumina sources results in an increase in the reactivity of nano-powders [13,14] and their ability to form Mg–Al–CO_3_ hydrotalcite-like compounds [8]. 

Due to the special lamellar structure, a new application has been found for hydrotalcite that enables refractory castable manufactures to use reactive nano- and micropowders of alumina (Al_2_O_3_) and magnesia (MgO) as alternative cementitious materials. Various authors highlighted that materials with “in situ” formed Mg–Al hydrotalcite-like phases exhibit higher mechanical strength of the green body than hydratable alumina-free refractory systems. This advantage is related to the fact that the spinel-like phase can be formed at lower temperatures [15,16,17]. There are many publications about the methods of obtaining hydrotalcite-like phases; it can be obtained through mechanochemical synthesis, sol–gel syntheses or hydrothermal precipitation [8,18,19,20]. Nevertheless, there are no published data concerning obtaining the hydrotalcite from a mixture of nanometric MgO and Al_2_O_3_ oxides. According to the MgO–Al_2_O_3_–H_2_O system at low temperatures, the products of the reaction of magnesium and alumina oxides with water are single or/and double hydroxides. Layered double hydroxides (LDHs) are represented by the general formula ${\left[Mg_{1-x}Al_{x}{\left(OH\right)}_{2}\right]}^{x+}{\left[{\left(A^{n-}\right)}_{\frac{x}{n}}\cdot yH_{2}O\right]}^{x-}$ (where A^n−^ is the exchangeable interlayer anion located between two LDH sheets and n- is a charge) [21,22]. The hydrotalcite structure is derived from the structure of brucite and the range of *x* can be varied, depending on literature sources, between 0.17 and 0.33 or 0.1 to 0.5 [23,24,25]. The main hydration product is crystalline, inorganic Mg_6_Al_2_(OH)_16_]CO_3_∙4H_2_O, which bonds together phases in the binder materials [9,10]. The structural units are made from stacks of octahedral sheets which have a positive charge. Due to substitution in Mg(OH)_2_ of divalent ions (Mg^2+^) with trivalent (Al^3+^) ones, the net positive charge occurs what is balanced by the interlayer anions. This structure is also stabilized by appropriate amounts of water, which are hydrogen-bonded to the interlayer anions or to the hydroxide layers [8,26].

The Mg–Al hydrotalcite-like phases present their thermal decomposition in a few steps, wherein the release of free-water and physically adsorbed CO_2_ takes place up to 200 °C while the inter-lamellar water may be withdrawn at 200–300 °C [15]. The dehydration process leads to the loss of crystallinity and shrinkage of the layer lattice dimensions which can indicate the segregation into Mg(OH)_2_ and Al-rich regions or the coexistence of MgO with hydrotalcite, as it has been suggested by MacKenzie, Taylor and Miyata [27,28,29]. The thermal decomposition at temperatures above 400 °C (hydrothermal conditions) or 1000 °C (aqueous solution of MgCl_2_ and AlCl_3_), depending on the experimental method, leads to obtaining first a series of metaphases forming a mixture of MgO and MgAl_2_O_4_ as a final product [21,30,31,32]. During the calcination process at intermediate temperatures, some transition aluminas (γ–Al_2_O_3_) or Al–MgO solid solutions were formed before spinel appeared as a final phase. The formation of this solid solution is associated with the substitution of magnesium ions with aluminum ions in the tetrahedral positions [32]. The IR spectrum of γ–Al_2_O_3_ presents a wide unresolved pattern with maximum absorbance around 800, 600 and 380 cm^−1^. According to the literature sources, the lowest energy band, which appears around 380 cm^−1^, can be assigned to AlO_4_ and AlO_6_ bending modes. Both wide structures, 600 and 800 cm^−1^, are related to AlO_6_ and AlO_4_ stretching, respectively [33,34,35]. At elevated temperatures, the final stable product of the hydrotalcite dehydration/dehydroxylation is spinel. Two types of spinels can be distinguished: normal and inverse spinels. In the normal, ideal spinel type, all 16 Al^3+^ ions are in octahedral coordination and all of eight Mg^2+^ ions are in tetrahedral coordination. The general formula can be written as (Mg_8_)[Al_16_]O_32_ or (Mg)[Al_2_]O_4_ for the cubic cell, where () and [] signify tetrahedral and octahedral sites, respectively. The inverse spinel may be described by the formula (Al)[MgAl])_4_ or (Al_8_)[Mg_8_Al_8_]O_32_. The difference in structure is that the Mg^2+^ and Al^3+^ ions occupy the octahedral sites in equal proportions. This inversion can be described as parameter γ, which is the fraction of octahedral sites occupied by Mg^2+^. In that case, the structural formula can be written as (Mg_1−2γ_Al_2γ_)[Mg_2γ_Al_2−2γ_]O_4_. The γ parameter of 0, $\frac{1}{2}$, and $\frac{1}{3}$ indicate: normal spinel, inverse spinel and completely random ions distribution, respectively [36].

Based on the presented above aspects, the aim of this work is to investigate the synthesis and thermal decomposition mechanisms of Mg–Al layered double hydroxide as a magnesia-alumina spinel precursor for cement-free corundum-spinel castables. For this purpose, the effect of time on the progress of hydration of nano-MgO–nano-Al_2_O_3_ blended paste structure was studied. On the other hand, the spinel formation in a smart nano-structured matrix was analyzed through the thermal transformation of the hydrotalcite-containing blended paste into spinel, being an effective ceramic bonding designed for no-cement corundum-spinel refractory castables. In summary, the combination of nano-powders of MgO and Al_2_O_3_ and water provides a cement and CaO-free binding system designed for alumina-based castables. The presented approach illustrates the direction of the chemical reaction within this system, which is slower or faster than within the other system containing, e.g., micropowders or colloidal suspensions, respectively. The Mg–Al layered double hydroxide-like phases formed are considered the main binding agents and precursors for the low-temperature synthesis/formation of the MgAl_2_O_4_ spinel phase. We propose state-of-the-art supplementary cementitious materials, representing an environmentally friendly alternative with strong potential for castables, since no CO_2_ is emitted during cement-free production processes.

## 2. Experimental Sections

### 2.1. Sample Preparation and Analytical Techniques

The synthesis of the Mg–Al–CO_3_ hydrotalcite-like phase was obtained by a curing process of the nano-MgO–nano-Al_2_O_3_ blended paste prepared with MgO nano-powder (<50 nm particle size, Aldrich, 99.8%) and Al_2_O_3_ nano-powder (13 nm particle size, Aldrich, 99.8%). For this purpose, the dry mix of nano-powders was weighted with the MgO:Al_2_O_3_ molar ratio of 1:1, homogenized and mixed with water, keeping the ratio of the weight of water to the weight of dry mix equal to 3. The sample in paste form was sealed in polyethylene bags and cured up to 49 days in a climatic chamber with the relative humidity maintained at 95% and a temperature of 50 °C. Two reference samples were prepared via the hydration of MgO nano-powder or Al_2_O_3_ nano-powder under the same curing conditions to achieve hydroxides for 28 days. To identify the blended paste phase changes corresponding to the curing time, X-ray diffraction (XRD), Fourier transform infrared spectroscopy (FT–IR) and thermal analysis, i.e., simultaneous thermogravimetry (TG), differential scanning calorimetry (DSC) and evolved gas analysis-mass spectrometry (EGA–MS), were used. Samples for the ex situ measurements were ground in acetone to discontinue the hydration after 24 h, 3 days, 14 days, 28 days and 49 days. The in situ high-temperature XRD (HT–XRD) measurement was applied to evaluate a complex sequence of dehydration, dehydroxylation, decarbonization of the fully-reacted blended paste and formation of a series of metaphases at elevated temperatures. The temperatures were suggested by the thermal analysis results.

As a next step, the fully-reacted (after 49 days of hydration) nano-MgO–nano-Al_2_O_3_ blended paste containing synthetic hydrotalcite was heated to 600, 700, 800, 900, 1000 and 1100 °C and maintained at a selected temperature for 10 h. The solid calcination products were characterized by ex situ XRD, FT–IR and ^27^Al MAS–NMR (solid-state magic-angle spinning nuclear magnetic resonance) measurements.

Furthermore, sintering of an equimolar mixture of MgO (Acros Organics, 98% MgO) and Al_2_O_3_ (Acros Organics, 98% Al_2_O_3_) micropowders in three-steps with intermediate grinding was applied with success for the synthesis of the stoichiometric spinel MgAl_2_O_4_ as reference material. The holding time was 10 h and the applied temperature 1700 °C. This reference material was investigated by NMR, FT–IR and XRD.

### 2.2. Measurement Conditions

The strength of the binder alternative to calcium aluminate cement containing MgO and Al_2_O_3_ micropowders was determined using standard tabular alumina, a 1:1 molar mixture of MgO and Al_2_O_3_ and water. A basic mixture of mortar was made using the mass proportions of binder (25 mas. %), standard tabular alumina (75 mas. %) and water. The mass proportion of 1 binder and 0.75 water (the water/binder mass ratio of 0.75) was applied for this purpose. The reference mortar containing calcium aluminate cement was also prepared with a water/cement mass ratio of 0.5.

The measurements of bending strength (modulus of rupture (MOR)) and cold crushing strength (CCS) of samples were taken after 3 days and 7 days of curing at room temperature. The bending strength was measured by a 3-point bending test using a specimen of 25 × 25 × 120 mm, a span of 75 mm and a test speed of 50 N/s (reference CAC-based mortar) or 10 N/s (MgO–Al_2_O_3_-based mortar). After the mechanical 3-point bending test, half of each sample was placed in the compression testing equipment in order to determine the CCS, and the loading speed was controlled at 2.4 kN/s (reference CAC-based mortar) or 50 N/s (MgO–Al_2_O_3_-based mortar). The thermal insulation properties (thermal conductivity *λ*, thermal diffusivity *a*, volumetric heat capacity C_v_) of both mortars were measured at room temperature by ISOMET 2114 working on a dynamic measurement principle. For this purpose, the prismatic samples with dimensions of 40 × 40 × 160 mm were cast from a basic mixture of mortars. The heat transport parameters determination was made 3 days after casting the mortar specimens.

Thermal analysis was carried out in airflow (50 mL∙min^−1^) at a heating rate of 10 °C∙min^−1^ using a Simultaneous Thermo Analyzer (STA) TG–DSC NETZSCH STA 449F5 Jupiter coupled to QMS 403 D Aëolos (Erich NETZSCH GmbH & Co. Holding KG, Selb, Germany). The provided sample mass ~20 mg was heated to 1000 °C in a corundum crucible. Alpha-Al_2_O_3_ was used as a standard substance.

The in-situ HT–XRD measurement was carried out at temperatures suggested by the thermal analysis results for a 2θ-range of 1–70° with a step size of 0.016° and a counting time per step of 4 s. The heating rate was 10 °C∙min^−1^, and upon reaching the temperature, it was kept constant for 3 h before the measurement at constant temperature (30, 100, 150, 200, 250, 350, 390, 450 and 500–1100 °C with a step of 100 °C) was carried out. The X’Pert Pro (PANalytical, Malvern PANalytical, Malvern, UK) diffractometer was used for this purpose.

The ex situ XRD measurement was carried out at room temperature using an X’Pert ProPANalytical X-ray diffractometer, with Cu Kα radiation, with 0.02° per step and a time of 3 s per step (2θ-range of 5°–90°). HighScore Plus software (Panalytical) with the PDF-2 database supported by the ICDD (The International Centre for Diffraction Data, Newtown Square, USA) was used for data analysis.

The FT–IR measurements were carried out using a Bruker Vertex 70v FT–IR spectrometer on pelletized ca. 0.5 wt.% powder samples in a KBr matrix. The IR bands were recorded at a spectral range of 400–4000 cm^−1^.

The ^27^Al MAS–NMR spectra were acquired using a BRUKER Avance III 400WB (9.4T) spectrometer, (BRUKER BioSpin, Rheinstetten, Germany).

## 3. Results and Discussion

### 3.1. Mechanism of Hydrotalcite Formation in the nano-MgO–nano-Al_2_O_3_ Blended Paste

**Figure 1:**
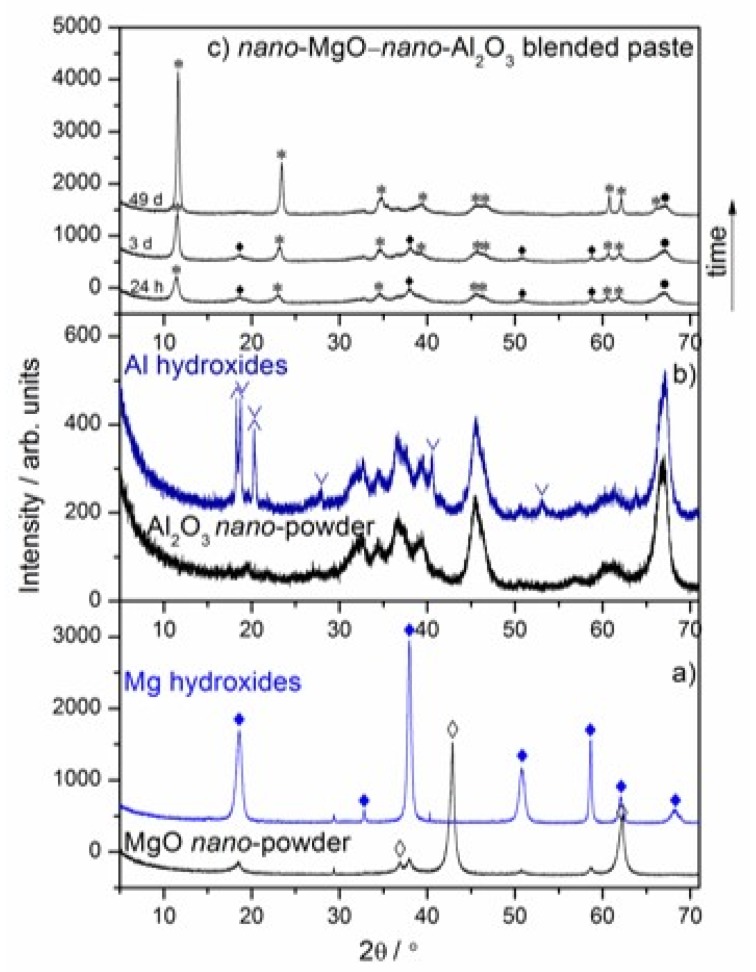
X-ray diffraction patterns of the reference of nano-MgO and synthesized nano-Mg(OH)_2_ (**a**), the reference nano-Al_2_O_3_ and synthesized hydrated nano-Al_2_O_3_ (**b**), the nano-MgO–nano-Al_2_O_3_ blended paste cured between 24 h and 49 days (**c**). ♦ Mg(OH)_2_ (JCPDS Card No. 98-007-9198), ◊ MgO (JCPDS Card No. 98–004–4927), * Mg_6_Al_2_CO_3_(OH)_16_·4H_2_O (JCPDS Card No. 00–022–0700), • Al_2_O_3_ (JCPDS Card No. 00–046–1131), ∨ Bayerite (JCPDS Card No. 98-002-6830), ∧ Gibbsite (JCPDS Card No. 00–007–0324).

**Figure 2:**
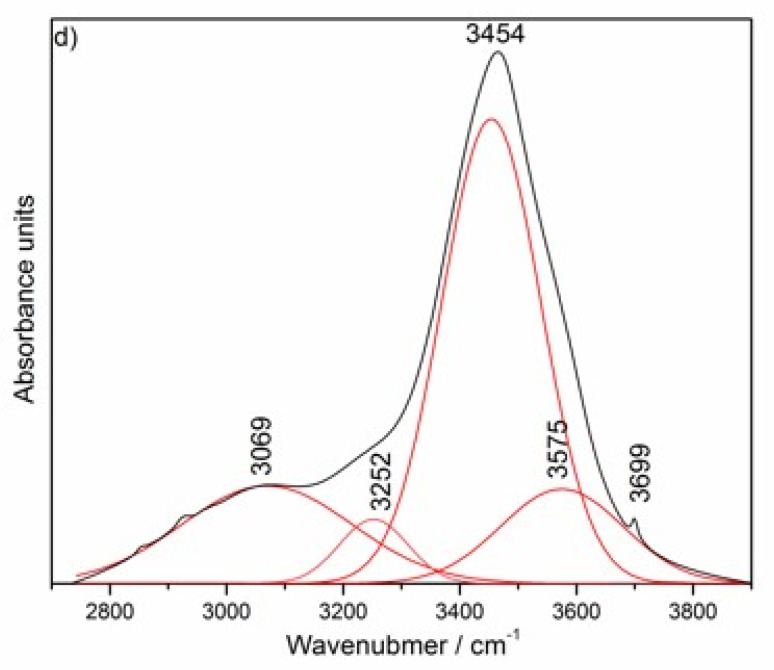

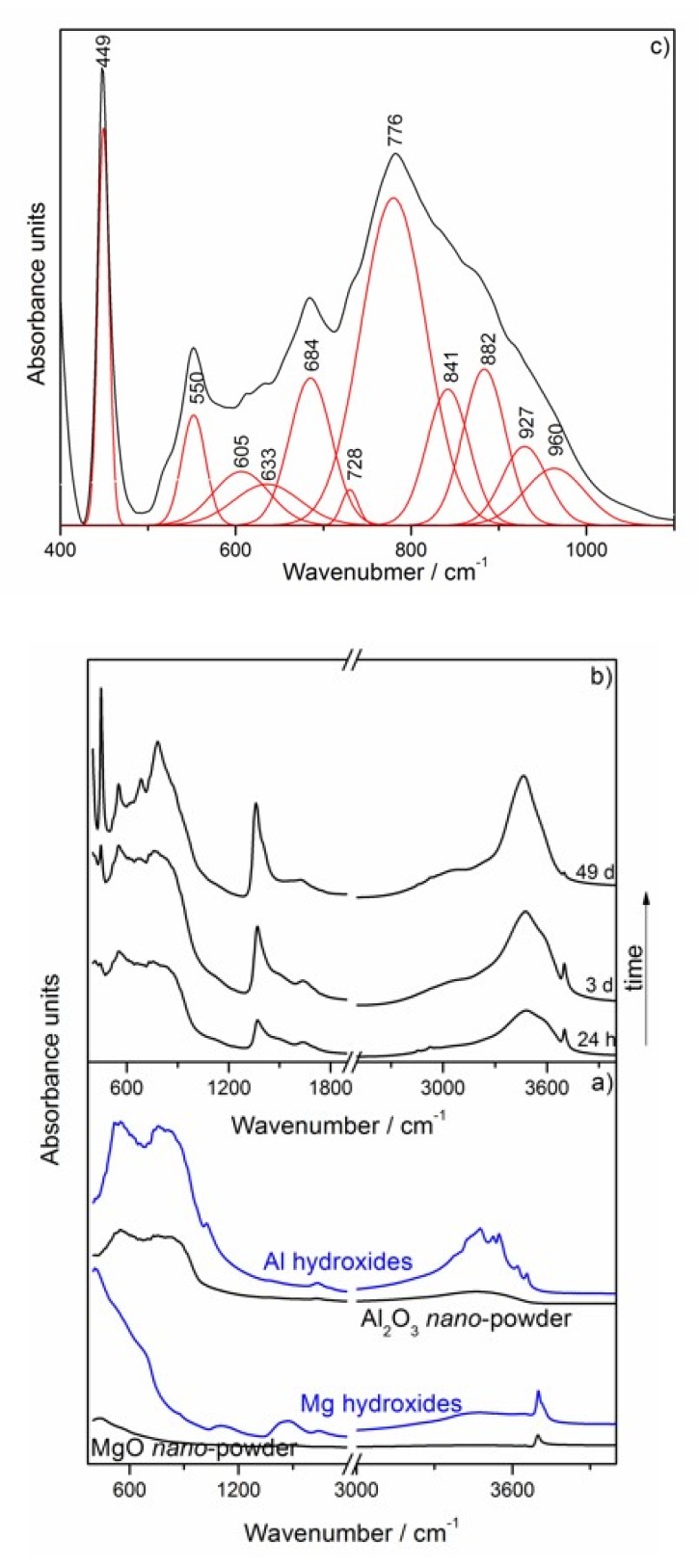
FT–IR spectra of the reference of nano-MgO and synthesized nano-Mg(OH)_2_, the reference nano-Al_2_O_3_ and synthesized hydrated nano-Al_2_O_3_ (**a**), the nano-MgO–nano-Al_2_O_3_ blended paste cured between 24 h and 49 days (**b**). Deconvoluted FT–IR spectrum of the nano-MgO–nano-Al_2_O_3_ blended paste at the age of 49 days in the range of 400–1100 cm^−1^ (**c**) and 2700-3800 cm^−1^ (**d**).

**Figure 3:**
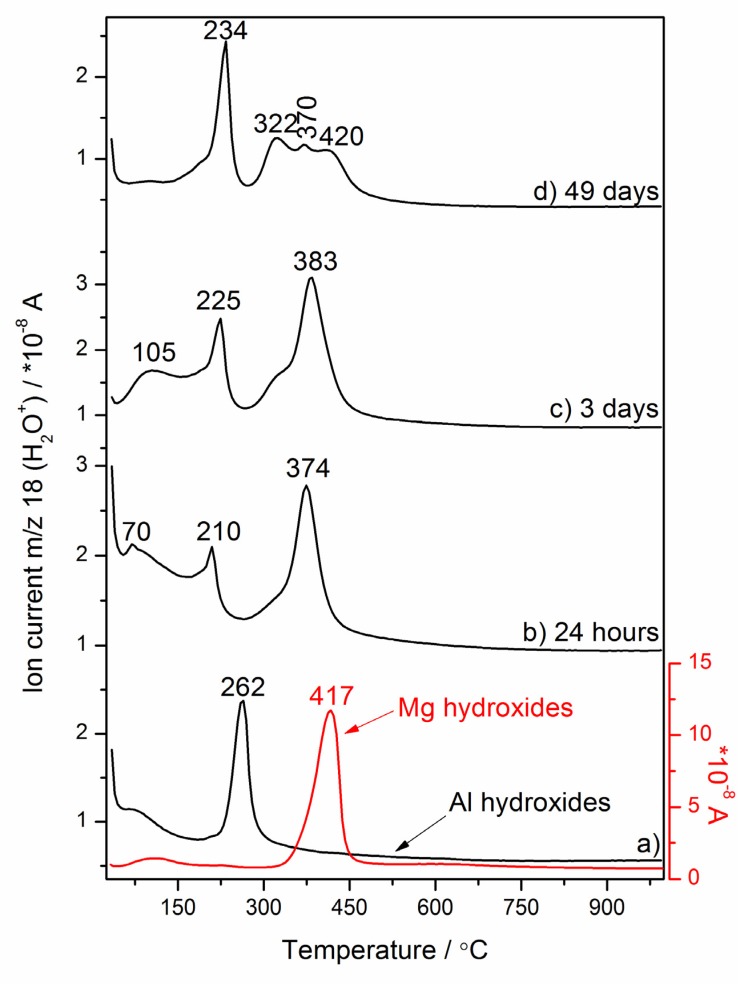
Evolution of the MS profile of the gaseous product (*m*/*z* = 18 (H_2_O^+^)) characteristic for the nano-MgO–nano-Al_2_O_3_ blended paste decomposition vs. the temperature for different curing times (**b**) 24 h, (**c**,**d**) 3–49 days and the reference Mg hydroxides and Al hydroxides obtained via hydration of MgO and Al_2_O_3_ nano-powders, respectively (**a**).

The time-dependent hydration behavior of the nano-MgO–nano-Al_2_O_3_ blended paste was investigated at the age of 24 h and 3–49 days by the following complementary methods: X-ray diffraction (XRD), Fourier transform infrared spectroscopy (FT–IR), differential scanning calorimetry and thermogravimetry analyses coupled with evolved gas mass spectrometry (DSC–TG–MS). The observations relevant to the specimens tested after each curing period are presented in Figure 1, Figure 2 and Figure 3. In Figure 1a,b, the X-ray diffraction patterns indicate the presence of the commercially available raw materials by a very large MgO peak at 42.920° (JCPDS Card No. 98–004–4927), an Al_2_O_3_ peak at 67.247° (JCPDS Card No. 00–046–1131) and their hydrated counterparts by an Mg(OH)_2_ peak at 37.981° (JCPDS Card No. 98–007–9198), Al(OH)_3_ peaks at 18.814° for bayerite (JCPDS Card No. 98–002–6830) and at 18.277° for gibbsite (JCPDS Card No. 00–007–0324). The given patterns will be useful for further monitoring the compositional changes within the nano-MgO–nano-Al_2_O_3_ blended paste. As can be recognized from Figure 1c, magnesium oxide and aluminum oxide nano-powders react almost immediately with water to form a magnesium-aluminum hydrotalcite compound, i.e., Mg_6_Al_2_CO_3_(OH)_16_·4H_2_O, during the initial 24 h curing period after wet homogenization. It was also shown that traces of initially formed Mg(OH)_2_ were consumed during hydration and more and more hydrotalcite-like compounds were formed. The time evolution of the main characteristic diffraction lines of Mg_6_Al_2_CO_3_(OH)_16_·4H_2_O from the XRD peak profiles into sharp (006), (0012), (0018), (0210), (1118), (0024), (223), (2026) and (1310) crystalline peaks are observed in the nano-MgO–nano-Al_2_O_3_ blended paste (JCPDS Card No. 00-022-0700) (Figure 1c). The XRD pattern of the nano-MgO–nano-Al_2_O_3_ blended paste shows the expected hydration product including mainly the magnesium-aluminum hydrotalcite compound, i.e., Mg_6_Al_2_CO_3_(OH)_16_·4H_2_O, which was formed after 49 days curing period. Nevertheless, traces of nano-Al_2_O_3_ powder were still present. 

The progress of hydrotalcite formation in the nano-MgO–nano-Al_2_O_3_ blended paste observed by FT–IR method confirmed previous results from XRD. The most characteristic features of the FT–IR spectra of all blended pastes (Figure 2b) is the gradual disappearance of the sharp band at ca. 3701 cm^−1^ as a result of the consumption of the initially formed, free Mg(OH)_2_ and the appearance of the broad band in the range of ca. 3000–3700 cm^−1^, mainly due to O-H groups present in metal hydroxide layers (hydroxyl stretching region). The interaction between Mg(OH)_2_ and nano-Al_2_O_3_ particles was demonstrated by a reduction in the spectral intensities of the alumina along with the curing time. According to our research, the outline IR spectrum of nanoscale-alumina raw material (Figure 2a) was close to the gamma aluminum oxide (γ–Al_2_O_3_) nanoparticles presented in [33]. The wide and unresolved spectrum extending from ca. 450 to 850 cm^−1^ is typical for a complex and disordered crystallographic structure of nanoscale-alumina. After the initial curing period of 24 h, no band related to Al–OH is seen in the spectrum, indicating no alumina hydroxides were formed, as shown by the comparison of two spectra for blended paste at early hydration age (24 h, Figure 2b) and hydrated nano-Al_2_O_3_ powder (Figure 2a). Since, in a layered structure of brucite, Mg was partially substituted by Al along with the curing time, the electroneutrality was attained by incorporation of carbonate anions in the interlayer space (ca. 1371 cm^−1^), where water molecules (ca. 1643 cm^−1^) were also located (Figure 2b). Figure 2c,d presents a deconvoluted FT–IR spectrum of the nano-MgO–nano-Al_2_O_3_ blended paste at the age of 49 days in the range of 400–1100 cm^−1^ (c) and 2700–3800 cm^−1^ (d). Peak separations were carried out using Gaussian deconvolution. This approach was implemented since the kinetic energy distribution molecules (functional groups) is described by the Gaussian distribution. In other words, Doppler broadening produces a Gaussian line shape due to the Gaussian distribution of molecular velocities. Hence, this approach can be used successfully to study functional groups present in the complex cementitious systems. The positions of infrared bands of hydrotalcite as the main phase formed within this sample are summarized in Table 1. In the region below ca. 1000 cm^−1^ the IR spectrum of uncalcined Mg–Al–CO_3_-hydrotalcite shows the absorption bands at 550, 776 cm^−1^ and 605 cm^−1^, which correspond to the ‘Al’–OH and ‘Mg’–OH translation modes, respectively. Another absorption near 449cm^−1^ is associated with M–O stretching vibrations (M = Mg and Al) in the octahedral host layers, whereas the more complex spectrum of the incompletely unreacted cementitious matrix contains substrates or transitory hydrates [10,37]. Moreover, strong out-of-plane symmetric deformation mode (ν_2_) and antisymmetric deformation mode (ν_4_) of hydrotalcite $CO_{3}^{2-}$ ions around 776 and 684 cm^−1^, were observed, respectively. The band at 1362cm^−1^ indicated the antisymmetric stretching vibration (ν_3_) of carbonate anion [37,38,39,40]. The bending vibration of interlayer water molecules (dO–H) [20,41] occurs as sharp bands at 1628 cm^−1^ and it can be concluded that interlayer water molecules are hydrogen-bonded to $CO_{3}^{2-}$ interlayer ions [8,10]. The broad and strong band around 3400 cm^−1^ is associated with the OH–metal vibration. There were the stretching vibrations of hydroxyl –OH groups attached to Mg and to both Mg and Al in brucite-like layers (OH–Mg_3_: 3575 cm^−1^ and OH–Mg_2_Al: 3454 cm^−1^, respectively) [42,43,44]. The band that appears at 3700 cm^−1^ is associated with the O–H stretching vibration in brucite Mg(OH)_2_. Other bands in this region should be attributed to the $CO_{3}^{2-}$–H_2_O bridging and H-bonded modes located at 3252 cm^−1^ and 3069 cm^−1^, respectively [8,10].

**Figure 4:**
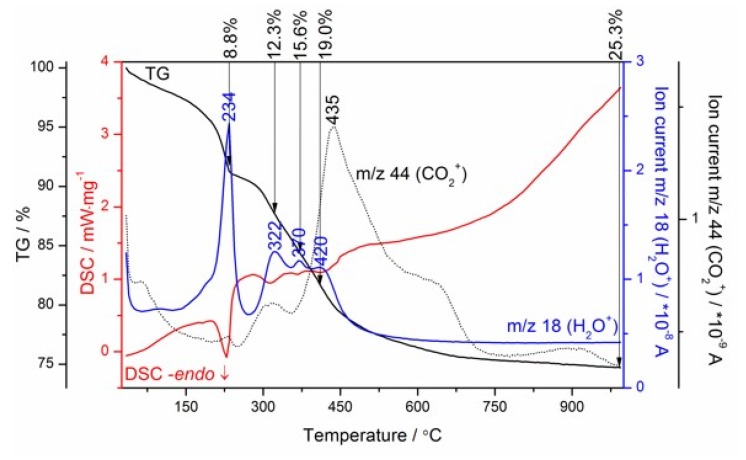
TG–DSC–MS curves of the Mg–Al–CO_3_ hydrotalcite-like phase formed in the MgO–Al_2_O_3_ blended paste after 49 days.

The gases evolved during heating the reference hydrated nano-MgO and nano-Al_2_O_3_ powders, and the nano-MgO–nano-Al_2_O_3_ blended paste cured between 24 h and 49 days, were analyzed using mass spectrometry (MS) (Figure 3b–d). Evolved gas analysis (EGA) detected an increase in ion current intensity for *m*/*z* = 18 (H_2_O^+^) produced by the electron impact ionization of water because of two reasons: H_2_O evolution monitored by the detection of fragments at *m*/*z* = 18 exhibits a parallel run to the DSC curves having local maximums at similar temperature values, and although the endothermic dehydration/dehydroxylation peaks are more sensitively detected by the MS than DSC curves, the EGA H_2_O profiles were only presented. As can be seen from Figure 3, different types of hydroxyl groups can exist in the nano-MgO–nano-Al_2_O_3_ blended paste and the pronounced structural rearrangements involving the hydroxyl groups can occur. This can be further supported by our previous findings [8] and other authors [45] that these four hydroxyl local environments are possible: Mg_3_–OH, Mg_2_Al–OH, MgAl_2_–OH and Al_3_–OH. The nature of the cation-hydroxyl bond and also the distribution of hydroxyl groups varied with hydration time (Figure 3b–d). This rearrangement is evident as a substitution of structural metal cations, like Mg^2+^ by cations of higher charge, and Al^3+^ in (Mg_3_)–OH–brucite-like sites, which initially becomes dehydroxylated at ca. 417 °C (Figure 3a). Along with the curing time, two hydroxyl local environments, i.e., (Mg_2_Al)–OH–hydrotalcite-like sites (~420 °C) and (Mg_3_)–OH–hydrotalcite-like sites (~322 °C), were progressively formed. Hence, the dehydration and dehydroxylation of hydrotalcite formed as a major product in the nano-MgO–nano-Al_2_O_3_ blended paste after 49 days of curing consecutively proceeds through the three main steps (Figure 4) involving the elimination of the interlayer structural water at ca. 234 °C and dehydroxylation overlapping with decarbonization processes at ca. 322 °C ((Mg_3_)–OH–hydrotalcite sites) and 420 °C ((Mg_2_Al)–OH–hydrotalcite sites). Besides, *m*/*z* = 18 exhibited an ion intensity peak at ca. 370 °C belonging to a possible residue of magnesium hydroxides ((Mg_3_)–OH–brucite-like sites). Typical DSC–TG heating curves showing hydrotalcite transition are found in the presentation in Figure 4. 

### 3.2. Heat Transport Parameters and Mechanical Properties

The results of the thermal conductivity, thermal diffusivity, volume heat capacity of two reference calcium aluminate (CAC)-containing and MgO–Al_2_O_3_-containing mortars are summarized in Table 2. All the heat transport parameters of mortars can be observed in the dependence of the applied hydraulic binder. These results represent important parameters for building designers, especially in the case of refractory castables. As can be clearly seen, all the heat transport parameters of MgO–Al_2_O_3_-based mortar are generally lower in comparison with the CAC-based mortar. The reduced thermal conductivity of MgO–Al_2_O_3_-based mortar cannot be viewed as beneficial since a low thermal conductivity was a barrier for sufficient thermal energy transfer from the materials to the environment. Hence, thermal shock resistance of refractories is increased by increasing the thermal conductivity, the CAC-based mortars should be considered at this stage as more beneficial. Nevertheless, both CAC-based and Mg–Al-based refractory castables after firing at elevated temperatures exceeding 1500 °C require further research.

Table 3 summarizes the mechanical properties of both the MgO–Al_2_O_3_-based and the reference CAC-based mortars. The mechanical properties of both cold crushing strength and bending strength of mortars as estimated by the procedure presented in Section 2.2 increased with the increase of the curing time. The presented results show important parameters including the so-called “green mechanical strength” of mortars after casting and curing. As can be clearly seen, both cold crushing strength and bending strength of the MgO–Al_2_O_3_-based mortar are generally lower in comparison with the CAC-based mortar. This is understandable as the CAC-based mortar contains mainly hydrates, i.e., compounds, that have in their structures chemically bonded water molecules, whereas the MgO–Al_2_O_3_-based mortar contains mainly an Mg(OH)_2_, M–A–H gel phase and unreacted starting constituents especially at the early stage of hydration and curing. Since the reactions within the MgO–Al_2_O_3_-H_2_O system are much slower than the corresponding reactions within the CaO-Al_2_O_3_ (CAC)–H_2_O system, an increase in strength with increasing time is slower as well.

### 3.3. Thermal Decomposition Mechanism of Mg–Al Layered Double Hydroxide as a Magnesia-Alumina Spinel Precursor

**Figure 5:**
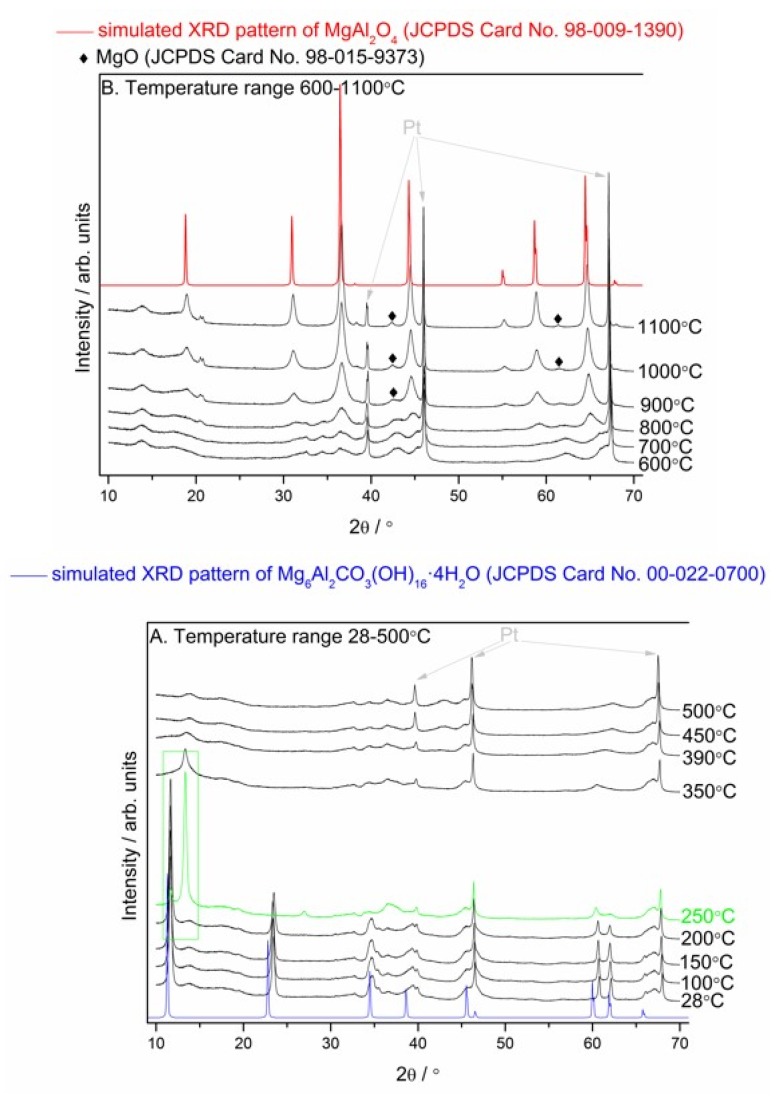
Evolution of HT–XRD patterns during the calcination of the Mg–Al layered double hydroxide from 28 to 500 °C (**A**) and from 600 to 1100 °C (**B**).

**Figure 6:**
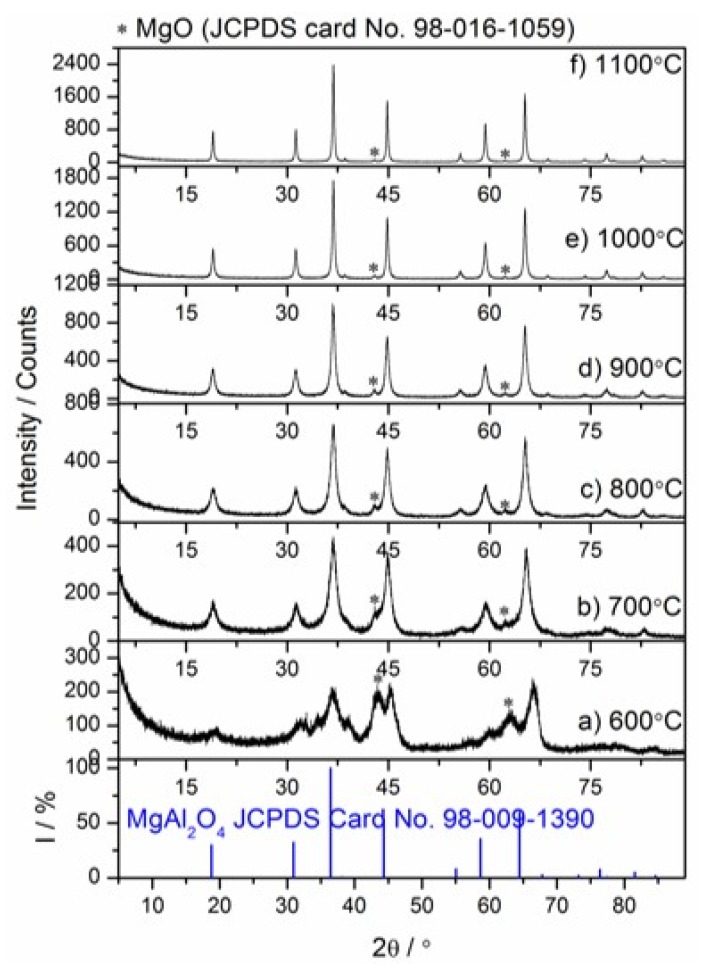
Low-temperature XRD patterns of the Mg–Al layered double hydroxide calcined in the temperature range 600–1100 °C (**a**–**f**).

**Figure 7:**
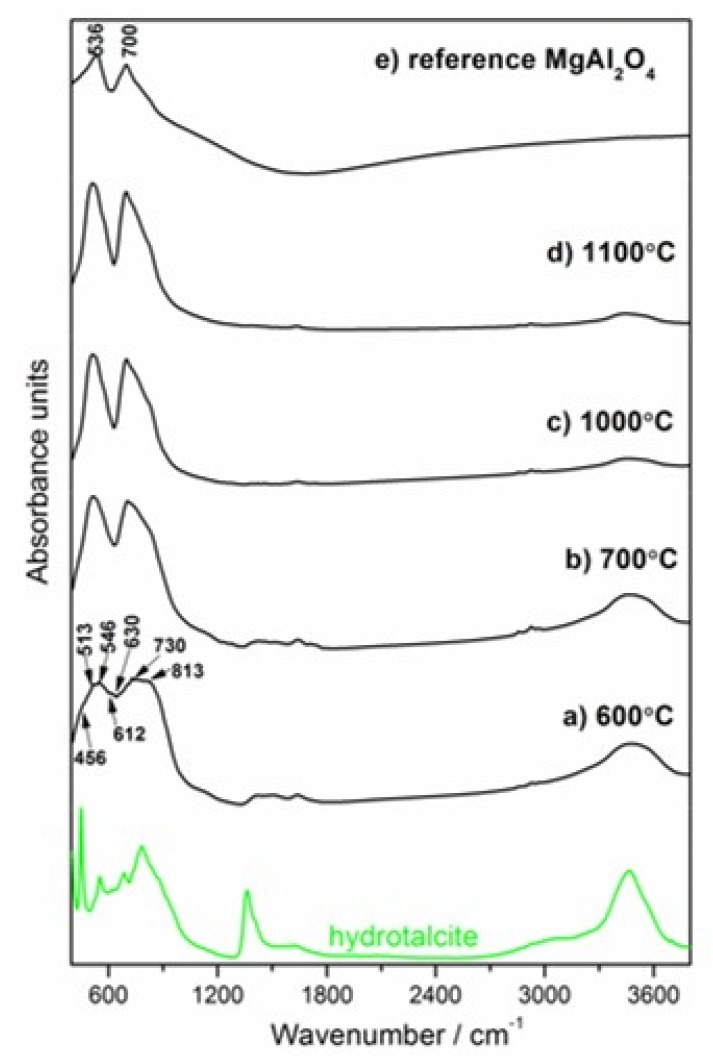
FT–IR spectra of the Mg–Al layered double hydroxide calcined in the temperature range 600–1100 °C (**a**–**d**). The green line is repeated from Figure 2 (hydrotalcite-containing paste hydrated for 49 days). The reference spectrum for MgAl_2_O_4_ synthesized via solid-state reaction at 1700 °C is presented in Figure 7e.

**Figure 8:**
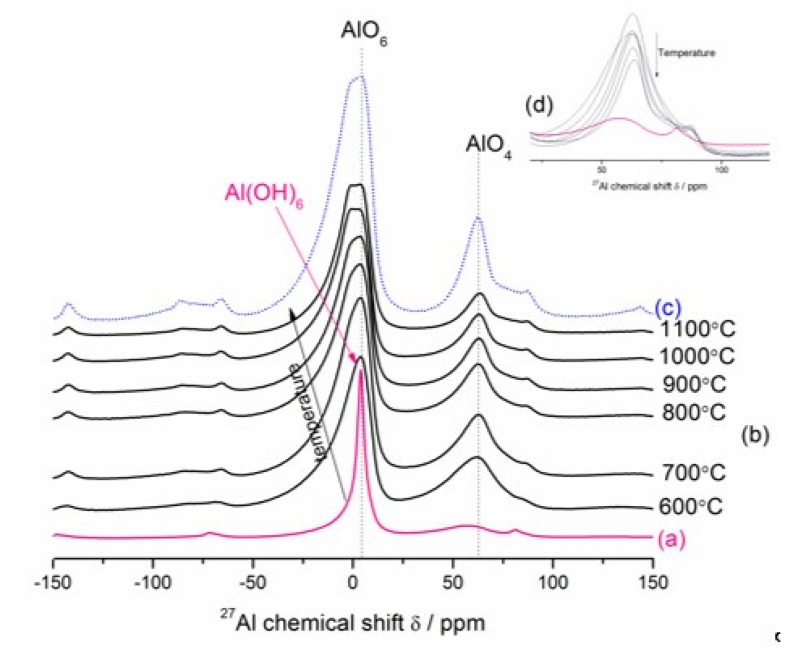
The room-temperature ^27^Al MAS–NMR spectra of the Mg–Al–CO_3_ hydrotalcite-like phase formed in the fully hydrated hardened MgO–Al_2_O_3_ blended paste (**a**), the Mg–Al layered double hydroxide calcined in the temperature range 600–1100 °C (**b**) and the reference spinel (**c**). Arrow in (**b**) means the changes according to the direction of increasing temperature from 600 to 1100 °C. (**d**) presents a line at about 62 ppm (tetrahedrally coordinated lattice Al^3+^).

The thermal decomposition mechanism of Mg_6_Al_2_CO_3_(OH)_16_∙4H_2_O and MgAl_2_O_4_ formation mechanism were analyzed by in situ HT–XRD and ex situ LT–XRD, FT–IR and NMR techniques. The temperatures for HT–XRD were suggested by the thermal analysis results. The relevant results are presented in Figure 5, Figure 6, Figure 7 and Figure 8.

#### 3.3.1. In Situ High-Temperature X-Ray Diffraction (HT–XRD) Studies of the Mg–Al layered Double Hydroxide

Figure 5a,b presents the evolution of HT–XRD patterns during the calcination of the Mg–Al layered double hydroxide from 28 to 1100 °C and simulated XRD patterns of Mg_6_Al_2_CO_3_(OH)_16_·4H_2_O (according to JCPDS Card No. 98–009–1390) and MgAl_2_O_4_ (according to JCPDS Card No. 98–009–1390). The XRD results pointed out the crystalline hydrotalcite as the major phase in the sample hydrated for 49 days (see the XRD pattern in Figure 1). An increase in the calcination temperature to 200 °C does not result in changes in the phase composition of the sample. A significant difference is observed when the temperature reaches 250 °C. At this calcination temperature, the characteristic peak belonging to the Mg–Al hydrotalcite (2θ = 11.6930°, JCPDS Card No. 00–022–0700) shifts toward a higher 2θ value (13.3934°). This shift of the diffraction peak indicates structural changes due to the interlamellar water release. During the dehydration process, loss of crystallinity and shrinkage of the layer lattice dimensions have been observed by other researchers [27,29,46,47]. At ca. 350–390 °C, there were no diffraction peaks belonging to the hydrotalcite while at 700 °C in XRD pattern appeared peaks which are in excellent agreement with simulated XRD pattern of MgAl_2_O_4_ (JCPDS Card No. 98–009–1390). The other low intense peaks present at 900 °C or lower temperatures are the major peaks of MgO which are broadened due to poor crystallinity or small crystallites, or both. Moreover, some peaks belonging to the Pt plate were unavoidable. 

#### 3.3.2. Ex-situ LT–XRD, FT–IR and NMR Investigations

The ex situ LT–XRD, FT–IR and NMR measurements provided insights into both dehydration/dehydroxylation of hydrotalcite and the mechanism of spinel formation from the thermal decomposition of hydrotalcite. The observations relevant to the specimens tested after heat treatment at 600 to 1100 °C are presented in Figure 6, Figure 7 and Figure 8. As can be observed in Figure 6a, the high and broad amorphous background of the XRD pattern of hydrotalcite calcined sample and some relatively weak XRD peaks confirm that full dehydration, dehydroxylation and decarbonization processes occurred at 600 °C. The amorphous character of hydrotalcite calcination products, probably spinel nuclei, transition alumina-type structures or Mg(Al)O mixed oxides, was also found in the other works [27,28]. These phases were too poorly crystalline to be detected by XRD. The half-width at half maximum of the peaks belonging to spinel, indicated by the reference pattern JCPDS Card No. 98–009–1390, is decreasing with the increasing calcination temperature from 700 to 1100 °C (Figure 6b–f). This indicates the significant increase in crystallinity, particle size of the spinel or both. It can be also noted that the amount of spinel phase in the samples increased with an increase in calcination temperature confirmed as an increase in XRD peak intensity. The appearance of crystalline undoped (Al-free) MgO is consistent with other literature sources [30,31].

As observed in the spectra of Figure 7b, the spectrum of the sample calcined at 700 °C presents characteristic bands of spinel, similar to the reference sample band around 536 and 700 cm^−1^ (Figure 7e) [48]. It can be noticed that spectra present a better resolved and more intense spinel structure as the calcination temperature increases (Figure 7a–d). The infrared spectrum of the sample calcined at 600 °C presents a wide, unresolved pattern extending from 380 to 1100 cm^−1^, with maximum absorbance at around 530 and 800 cm^−1^ (Figure 7a). The calcination process at 600 °C induced dehydration, dehydroxylation and decarbonation which lead to the formation of mixed oxides of Al_2_O_3_ and MgO or a spinel-like precursors phase. The peaks around 730–630 cm^−1^ (v_4_–MgO or Al–O) and around 513 cm^−1^ (v_5_–Mg–O or Al–O) can be attributed to the presence of Mg–O and Al–O bands [49]. Additionally, the band at 546 cm^−1^ is associated with the presence of MgO. The other two bands (612 and 813 cm^−1^) were related to AlO_6_ and AlO_4_ stretching, respectively, thus confirming the presence of γ–Al_2_O_3_ in the sample [33]. According to another literature source [50], the band at 456 cm^−1^ was due to the presence of the Mg–O–Al bond that was originally present in the Mg/Al hydrotalcite. The similarity in the shape of the IR spectrum of the sample calcined at 600 °C to the γ–Al_2_O_3_ IR curve may indicate the appearance of the gamma-like structure phase.

The ^27^Al MAS–NMR spectroscopy was used to characterize cation distribution in the magnesium aluminate spinel and its precursor on an atomic level. Generally, in the spinel structure (AB_2_O_4_), the cations can be occupied by tetrahedral and octahedral coordination. In a normal spinel, all divalent ions (Mg^2+^) are located in A-type sites of the lattice and all trivalent ions (Al^3+^) are located in B-type sites of the lattice. The distribution of cations over these sites is expressed as (A)^tet^(B_2_)^oct^O_4_. *Inverse* spinels exhibit the configuration (B)^tet^(AB)^oct^O_4_, where the A^II^ ions occupy the octahedral voids, whereas one-half of B^III^ ions occupy the tetrahedral voids and the other half occupy octahedral sites. Nevertheless, most spinels show a degree of disorder between these two end members, with formula presented as (A_1−x_B_x_)^tet^(B_2−x_A_x_)^oct^O_4_, where *x* is the so-called degree of disorder [51,52]. Figure 8 shows room-temperature ^27^Al MAS–NMR spectra of the Mg–Al–CO_3_ hydrotalcite-like phase formed in the fully hydrated hardened MgO–Al_2_O_3_ blended paste after 49 days (a), the Mg–Al layered double hydroxide heat-treated in the temperature range 600–1100 °C (b) and the reference spinel prepared via three-step sintering of equimolar mixtures of MgO and Al_2_O_3_ micropowders at 1700 °C (c). The ^27^Al MAS–NMR spectrum of the sample before calcination (Figure 8a) shows a relatively narrow resonance peak at a chemical shift, δ, of ~4.0 ppm, which represents the octahedral coordination (0–10 ppm) of Al, Al(OH)_6_, in the hydrotalcite structure [53]. The trend observed from Figure 8b,c is the evolution of structural changes of fully dehydrated/dehydroxylated samples during treatment in the temperature range of 600–1100 °C to proceed in the direction of primary spinel crystallization. The interesting observation is that the increase in calcination temperature brings about both a noticeable broadening and a shift (toward more negative chemical shifts) of the AlO_6_ spectral line (see Figure 8b), implying a change in the local atomic environments of Al^3+^ ions due to some AlO_6_ deformation [54]. The spectra were shifted in the direction of the arrow. The spectra of calcined samples also consist of broad and small resonance at ca. 62 ppm (inset figure) in the region characteristic of tetrahedrally coordinated aluminum (40–80 ppm) [27,53,55,56] formed initially at temperatures below 600 °C. The appearance of tetrahedral Al in the fully dehydrated/dehydroxylated samples and its decreasing behavior with increasing calcination temperature from 600 to 1100 °C (Figure 8b,d) seems to have three overlapping reasons; one being the conversion of several possible metastable (transition) aluminas polymorphs (probably γ–Al_2_O_3_ or transition alumina-type structure [27,28,54,56,57,58,59,60] considered also as a mixed tetrahedral-octahedral Al_2_O_3_ phase [61] formed initially below 600 °C to the most stable (six-coordinated) corundum phase [59], the second being the migration of Al^3+^ ions from the periclase structure probably containing Al^3+^ in solid solution (unstable MgO-like phase or periclase Mg(Al)O) [54,62,63,64,65] or the other being the secondary spinel formation via reaction of a high surface area MgO–Al_2_O_3_ mixed oxides [30,47,54,66,67] (or an amorphous mixed-phase oxide of nominal composition MgAlO_x_ [62]) derived from hydrotalcite, in addition to the primary spinel derived from hydrotalcite. Note also that all the transition aluminas contain Al^3+^ in both tetrahedral and octahedral sites [68], whereas Al^3+^ in the periclase structure can be tetrahedrally [27,65,67,69,70,71] or octahedrally [54,56,63,72,73,74] coordinated with oxygen. Simultaneously, the resonance centered at ca. 4 ppm is due to octahedrally coordinated aluminum in spinel, aluminas and periclase structures. Hence, the exact nature of such behaviors is not clear, since it is very difficult to distinguish between MgAl_2_O_4_, Al_2_O_3_ and MgO rock salt-like structure, with the Al^3+^ cations evenly distributed. Finally, the ^27^Al MAS–NMR spectra of sample calcined at 1100 °C (Figure 8b) become more similar in character to the reference spectrum of magnesium aluminate disordered/inverse spinel powder synthesized at 1700 °C (Figure 8c), which exhibits two well-resolved peaks [51] in the region characteristic of tetrahedrally coordinated aluminum (chemical shift δ ≈ 62 ppm) and octahedrally coordinated aluminum (δ ≈ 4 ppm). Thus, a mixture of well-crystallized MgO and MgAl_2_O_4_ as a final product of hydrotalcite thermolysis was obtained, as reported earlier by Stanimirova et al. [30,31], Valente et al. [64], Occelli et al. [47] and Auerbach et al. [75].

## 4. Conclusions

According to the current research, many certain conclusions can be drawn:

1. A smart nano-structured matrix containing manly hydrotalcite Mg_6_Al_2_CO_3_(OH)_16_·4H_2_O designed for no-cement corundum–spinel refractory castables is developed through the hydration of equimolar mixture of MgO and Al_2_O_3_ nano-powders.

2. Hydrotalcite is formed through the disappearance of initially formed Mg(OH)_2_. The (Mg_3_)–OH–brucite sites formed initially within the magnesia-alumina hydrating blended paste were replaced with (Mg_2_Al)–OH and (Mg_3_)–OH hydrotalcite sites with different thermal stability.

3. Thermal decomposition of a nano-structured matrix containing mainly hydrotalcite was a complex sequence of dehydration, dehydroxylation and decarbonization and this finally led to the formation of inverse spinel MgAl_2_O_4_ and periclase MgO through many intermediate stages containing the mixed tetrahedral-octahedral Al-rich phase and MgO-like structure. 

4. Important changes in oxygen coordination polyhedra around Al^3+^ involving the decrease in the amount of tetrahedral AlO_4_ units in the dehydrated-dehydroxylated hydrotalcite occurred in sample heated within the temperature range of between 600 and 1100 °C.

5. Hence, hydrotalcite was considered as a precursor of ultrafine spinel that exhibits increased crystallinity with increased processing temperature.

6. An inverse spinel MgAl_2_O_4_ was formed at relatively low temperature i.e., 700 °C.

7. The primary spinel was formed directly through the dehydration/dehydroxylation of hydrotalcite Mg_6_Al_2_CO_3_(OH)_16_∙4H_2_O.

8. Secondary spinel was formed through the solid-state reaction between other decomposition products MgO and Al_2_O_3_.

9. All presented results were supported by in situ HT–XRD and ex situ LT–XRD, FT–IR, DSC–TG–EGA(MS) and ^27^Al MAS–NMR examinations.

10. A novelty of the present work consists of the Mg–Al layered double hydroxide-like phases formed within the nano-MgO–nano-Al_2_O_3_ blended paste as the main binding agents and precursors for the low-temperature formation of the MgAl_2_O_4_ spinel phase.

## Figures and Tables

**Table 1:** The positions of infrared bands of the nano-MgO–nano-Al_2_O_3_ blended paste at the age of 49 days.

Uncalcined Mg–Al–CO_3_–Hydrotalcite Band Positions [cm^−1^]		Assignment
Bands Presented in this Work	Reference Bands	
449	447, 449 [8,10,37]	M–O stretching and M–OH bending vibrations (M = Mg, Al) in the octahedral host layers
550	552, 557 [8,37]	Al–OH translation modes
605	629 [8,10]	Mg–OH translation modes
684	667, 683 [8,10]	Antisymmetric deformation mode (ν_4_) of hydrotalcite $CO_{3}^{2-}$ ions
776	783 or 772, 783 [8,10,37,38]	Strong out-of-plane symmetric deformation mode (ν_2_) of hydrotalcite $CO_{3}^{2-}$ ions or Al–OH translation modes
1362	1358, 1365 [8,10,39]	Antisymmetric stretching vibration (ν_3_) of carbonate anion
1628	1622, 1655 [10,39]	Bending vibration of interlayer water molecules (dO–H)
3069	3045 [10,42]	H-bonded modes
3252	3250 [8,10,40,42]	$CO_{3}^{2-}-H_{2}O$ bridging mode
3454	3450 [8,10]	The stretching vibrations of hydroxyl –OH groups attached to both Mg and Al in brucite-like layers (OH–Mg_2_Al)
3575	3546 [8,10]	The stretching vibrations of hydroxyl –OH groups attached to Mg in brucite-like layers (OH–Mg_3_)
3700	3700 [10]	O–H stretching vibration in brucite

**Table 2:** Heat transport parameters of mortars.

Mortar	Heat Transport Parameters		
	Thermal Conductivity *λ* W/(mK)	Thermal Diffusivity *a* 10^6^ J/(m^3^K)	Volume Heat Capacity C_v_ 10^−6^ m^2^/s
CAC	2.584	1.739	1.486
MgO–Al_2_O_3_	2.063	1.546	1.335

**Table 3:** Mechanical properties of mortars.

Mortar	Time of Curing	Cold Crushing Strength/MPa	Bending Strength/MPa
CAC	3 days	81.33	10.59
	7 days	101.82	13.52
MgO–Al_2_O_3_	3 days	1.26	0.50
	7 days	1.54	0.74

## References

[1] Ghosh S., Majumdar R., Sinhamahapatra B.K., Nandy R.N., Mukherjee M., Mukhopadhyay S. (2003). Microstructures of refractory castables prepared with sol-gel additives. Ceram. Int..

[2] Nouri-Khezrabad M., Braulio M.A.L., Pandolfelli V.C., Golestani-Fard F., Rezaie H.R. (2013). Nano-bonded refractory castables. Ceram. Int..

[3] Mukhopadhyay S., Ghosh S., Mahapatra M.K., Mazumder R., Barick P., Gupta S., Chakraborty S. (2002). Easy-to-use mullite and spinel sols as bonding agents in a high-alumina based ultra low cement castable. Ceram. Int..

[4] Zhang Y., Li Y., Xu Y., Sang S., Jin S. (2017). Enhanced formation of magnesium silica hydrates (M-S-H) using sodium metasilicate and caustic magnesia in magnesia castables. Ceram. Int..

[5] Nied D., Enemark-Rasmussen K., L’Hopital E., Skibsted J., Lothenbach B. (2016). Properties of magnesium silicate hydrates (M-S-H). Cem. Conc. Res..

[6] Esteban-Cubillo A., Pina-Zapardiel R., Moya J.S., Barba M.F., Pecharromán C. (2008). The role of magnesium on the stability of crystalline sepiolite structure. J. Eur. Ceram. Soc..

[7] Ghanbari Ahari K., Sharp J.H., Lee W.E. (2002). Hydration of refractory oxides in castable bond systems-I: Alumina, magnesia, and alumina-magnesia mixtures. J. Eur. Ceram. Soc..

[8] Madej D. (2018). Examination of dehydration and dehydroxylation of synthetic layered (oxy)hydroxides through thermal analysis (TG-DSC-EGA-MS) and a discussion to the second Pauling’s rule. Inorg. Chim. Acta.

[9] Madej D., Prorok R., Wiśniewska K. (2018). An experimental investigation of hydration mechanism of the binary cementitious pastes containing MgO and Al_2_O_3_ micro-powders. J. Therm. Anal. Calorim..

[10] Madej D. (2017). Size-dependent hydration mechanism and kinetics for reactive MgO and Al_2_O_3_ powders with respect to the calcia-free hydraulic binder systems designed for refractory castables. J. Mater. Sci..

[11] Zhang P., Chen A., Ding D., Gao S., Liu X., Ye G., Liao G. (2019). Trace nanoscale Al_2_O_3_ in Al_2_O_3_-MgAl_2_O_4_ castable for improved thermal shock performance. Ceram. Int..

[12] dos Anjos R.D., Ismael M.R., de Oliveira I.R., Pandolfelli V.C. (2008). Workability and setting parameters evaluation of colloidal silica bonded refractory suspensions. Ceram. Int..

[13] Tang X., Guo L., Chen C., Liu Q., Li T., Zhu Y. (2014). The analysis of magnesium oxide hydration in three-phase reaction system. J. Solid. State. Chem..

[14] Ma W., Brown P.W. (1999). Mechanisms of reaction of hydratable aluminas. J. Am. Cer. Soc..

[15] dos Santos T., Pinola F.G., Luz A.P., Pagliosa C., Pandolfelli V.C. (2018). Al_2_O_3_-MgO refractory castables with enhanced explosion resistance due to in situ formation of phases with lamellar structure. Ceram. Int..

[16] Ye G., Troczynski T. (2005). Effect of magnesia on strength of hydratable alumina-bonded castable refractories. J. Mater. Sci..

[17] Salomão R., Pandolfelli V.C. (2009). The role of hydraulic binders on magnesia containing refractory castables: Calcium aluminate cement and hydratable alumina. Ceram. Int..

[18] Zhang X., Li S. (2013). Mechanochemical approach for synthesis of layered double hydroxides. Appl. Surf. Sci..

[19] Chubar N., Gerda V., Megantari O., Mičušík M., Omastova M., Heister K., Man P., Fraissard J. (2013). Applications versus properties of Mg-Al layered double hydroxides provided by their syntheses methods: Alkoxide and alkoxide-free sol-gel syntheses and hydrothermal precipitation. Chem. Eng. J..

[20] Wang J., Wei Y., Yu J. (2013). Influences of polyhydric alcohol co-solvents on the hydration and thermal stability of MgAl-LDH obtained via hydrothermal synthesis. Appl. Clay Sci..

[21] Komlev A.A., Gusarov V.V. (2011). Mechanism of the nanocrystals formation of the spinel structure in the MgO-Al_2_O_3_-H_2_O system under the hydrothermal conditions. Russ. J. Gen. Chem..

[22] Qu J., Zhang Q., Li X., He X., Song S. (2016). Mechanochemical approaches to synthesize layered double hydroxides: A review. Appl. Clay Sci..

[23] Gabrovska M., Edreva-Kardjieva R., Angelov V., Crişan D., Millet J.-M.M. (2007). Mg-Al and Mg-In oxide compounds as catalyst components for the oxidative dehydrogenation of propane. Part II Characterization of the calcined materials. Rev. Roum. Chim..

[24] Xu X., Li D., Song J., Lin Y., Lv Z., Wei M., Duan X. (2010). Synthesis of Mg-Al-carbonate layered double hydroxide by an atom-economic reaction. Particuology.

[25] Kovanda F., Jindova E., Doušová B., Kolouskova S. (2009). Layered double hydroxides intercalated with organic anions and their application in preparation of LDH/polymer nanocomposites. Acta Geodyn. Geomater..

[26] Oh J.-M., Hwang S.-H., Choy J.-H. (2002). The effect of synthetic conditions on tailoring the size of hydrotalcite particles. Solid State Ion..

[27] MacKenzie K.J.D., Meinhold R.H., Sherriff B.L., Xu Z. (1993). ^27^Al and ^25^Mg solid-state magic-angle spinning nuclear magnetic resonance study of hydrotalcite and its thermal decomposition sequence. J. Mater. Chem..

[28] Miyata S. (1980). Physico-chemical properties of synthetic hydrotalcites in relation to composition. Clay Clay Miner..

[29] Taylor H.F.W. (1973). Crystal structures of some double hydroxide minerals. Mineral. Mag..

[30] Stanimirova T., Piperov N., Petrova N., Kirov G. (2004). Thermal evolution of Mg-Al-CO_3_ hydrotalcites. Clay Miner..

[31] Stanimirova T., Vergilov I., Kirov G., Petrova N. (1999). Thermal decomposition products of hydrotalcite-like compounds: Low-temperature metaphases. J. Mater. Sci..

[32] Sato T., Kato K., Endo T., Shimada M. (1986). Preparation and chemical properties of magnesium aluminium oxide solid solutions. React. Solid..

[33] Boumaza A., Favaro L., Lédion J., Sattonnay G., Brubach J.B., Berthet P., Huntz A.M., Roy P., Tétot R. (2009). Transition alumina phases induced by heat treatment of boehmite: An X-ray diffraction and infrared spectroscopy study. J. Solid State Chem..

[34] Rinaldi R., Schuchardt U. (2005). On the paradox of transition metal-free alumina-catalyzed epoxidation with aqueous hydrogen peroxide. J. Catal..

[35] Krishna Priya G., Padmaja P., Warrier K.G.K., Damodaran A.D., Aruldhas G. (1997). Dehydroxylation and high temperature phase formation in sol-gel boehmite characterized by Fourier transform infrared spectroscopy. J. Mater. Sci. Lett..

[36] Mo S.-D., Ching W.Y. (1996). Electronic structure of normal, inverse, and partially inverse spinels in the MgAl_2_O_4_ system. Phys. Rev. B.

[37] Li B., Zhang Y., Zhou X., Liu Z., Liu Q., Li X. (2016). Different dye removal mechanisms between monodispersed and uniform hexagonal thin plate-like MgAl–$CO_{3}^{2-}$–LDH and its calcined product in efficient removal of Congo red from water. J. Alloys Compd..

[38] Kloprogge J.T., Frost R.L. (1999). Infrared emission spectroscopic study of the thermal transformation of Mg-, Ni- and Co-hydrotalcite catalysts. Appl. Catal. A General.

[39] Kloprogge J.T., Frost R.L. (1999). Fourier transform infrared and Raman spectroscopic study of the local structure of Mg-, Ni-, and Co-hydrotalcites. J. Solid State Chem..

[40] Tao Q., Reddy B.J., He H., Frost R.L., Yuan P., Zhu J. (2008). Synthesis and infrared spectroscopic characterization of selected layered double hydroxides containing divalent Ni and Co. Mater. Chem. Phys..

[41] Mora M., López M.I., Jiménez-Sanchidrián C., Ruiz J.R. (2011). Study of organo-hybrid layered double hydroxides by medium and near infrared spectroscopy. Spectrochim. Acta. A.

[42] Zhang J., Su H., Zhou J., Qian G., Xu Z., Xi Y., Xu Y., Theiss F.L., Frost R. (2013). Mid- and near-infrared spectroscopic investigation of homogeneous cation distribution in Mg_x_- Zn_y_Al_(x+y)/2_-layered double hydroxide (LDH). J. Colloid Interface Sci..

[43] Sideris P.J., Nielsen U.G., Gan Z., Grey C.P. (2008). Mg/Al ordering in layered double hydroxides revealed by multinuclear NMR spectroscopy. Science.

[44] Kloprogge J.T., Hickey L., Frost R.L. (2004). The effects of synthesis pH and hydrothermal treatment on the formation of zinc aluminium hydrotalcites. J. Solid State Chem..

[45] Nishimura S., Takagaki A., Ebitani K. (2013). Characterization, synthesis and catalysis of hydrotalcite-related materials for highly efficient materials transformations. Green Chem..

[46] Pesic L., Salipurovic S., Markovic V., Vueselic D., Kagunya W., Jones W. (1992). Thermal characteristics of a synthetic hydrotalcite-like mineral. J. Mater. Chem..

[47] Occelli M.L., Olivier J.P., Auroux A., Kalwei M., Eckert H. (2003). Basicity and porosity of a calcined hydrotalcite-type material from nitrogen porosimetry and adsorption microcalorimetry methods. Chem. Mater..

[48] Tarte P. (1967). Infra-red spectra of inorganic aluminates and characteristic vibrational frequencies of AlO_4_ tetrahedra and AlO_6_ octahedra. Spectrochim. Acta A Mol. Spectrosc..

[49] Zeng H.Y., Feng Z., Deng X., Li Y.Q. (2008). Activation of Mg-Al hydrotalcite catalysts for transesterification of rape oil. Fuel.

[50] Santosa S.J., Kunarti E.S. (2008). Karmanto. Synthesis and utilization of Mg/Al hydrotalcite for removing dissolved humic acid. App. Surface Sci..

[51] Šepelák V., Indris S., Bergmann I., Feldhoff A., Becker K.D. (2006). Nonequilibriumcation distribution in nanocrystalline MgAl_2_O_4_ spinel studied by ^27^Al magic-angle spinning NMR. Solid State Ion..

[52] Harris R.K., Wasylishen R.E., Duer M.J. (2009). NMR Crystallography.

[53] Park T.-J., Choi S.-S., Kim Y. (2009). ^27^Al solid-state NMR structural studies of hydrotalcite compounds calcined at different temperatures. Bull. Korean Chem. Soc..

[54] Shylesh S., Kim D., Gokhale A.A., Canlas C.G., Struppe J.O., Ho C.R., Jadhav D., Yeh A., Bell A.T. (2016). Effects of composition and structure of Mg/Al oxides on their activity and selectivity for the condensation of methyl ketones. Ind. Eng. Chem. Res..

[55] Béres A., Pálinkó I., Bertrand J.-C., Nagy J.B., Kiricsi I. (1997). Dehydration-rehydration behaviour of layered double hydroxides: A study by X-ray diffractometry and MAS NMR spectroscopy. J. Mol. Struct..

[56] Prescott H.A., Li Z.-J., Kemnitz E., Trunschke A., Deutsch J., Lieske H., Auroux A. (2005). Application of calcined Mg-Al hydrotalcites for Michael additions: An investigation of catalytic activity and acid-base properties. J. Catal..

[57] Lopes T.R., Gonçalves G.R., de Barcellos E., Schettino M.A., Cunha A.G., Emmerich F.G., Freitas J.C.C. (2015). Solid state ^27^Al NMR and X-ray diffraction study of alumina-carbon composites. Carbon.

[58] O’Dell L.A., Savin S.L.P., Chadwick A.V., Smith M.E. (2007). A ^27^Al MAS NMR study of a sol-gel produced alumina: Identification of the NMR parameters of the θ-Al_2_O_3_ transition alumina phase. Solid State Nucl. Magn..

[59] Chagas L.H., De Carvalho G.S.G., San Gil R.A.S., Chiaro S.S.X., Leitão A.A., Diniz R. (2014). Obtaining aluminas from the thermal decomposition of their different precursors: An ^27^Al MAS NMR and X-ray powder diffraction studies. Mater. Res. Bull..

[60] Jackson S.D., Hargreaves J.S.J. (2008). Metal Oxide Catalysis.

[61] vanBokhoven J.A., Roelofs J.C.A.A., de Jong K.P., Koningsberger D.C. (2001). Unique structural properties of the Mg-Al hydrotalcite solid base catalyst: An in situ study using Mg and Al K-Edge XAFS during calcination and rehydration. Chem. Eur. J..

[62] Gao Y., Zhang Z., Wu J., Yi X., Zheng A., Umar A., O’Hare D., Wang Q. (2013). Comprehensive investigation of CO_2_ adsorption on Mg-Al-CO_3_ LDH-derived mixed metal oxides. J. Mater. Chem. A..

[63] Ono Y., Hattori H. (2011). Solid Base Catalysis.

[64] Valente J.S., Sánchez-Cantú M., Lima E., Figueras F. (2009). Method for large-scale production of multimetallic layered double hydroxides: Formation mechanism discernment. Chem. Mater..

[65] Takehira K., Kawabata T., Shishido T., Murakami K., Ohi T., Shoro D., Honda M., Takaki K. (2005). Mechanism of reconstitution of hydrotalcite leading to eggshell-type Ni loading on Mg-Al mixed oxide. J. Catal..

[66] Valente J.S., Pfeiffer H., Lima E., Prince J., Flores J. (2011). Cyanoethylation of alcohols by activated Mg-Al layered double hydroxides: Influence of rehydration conditions and Mg/Al molar ratio on Brönsted basicity. J. Catal..

[67] Díez V.K., Apesteguía C.R., Di Cosimo J.I. (2003). Effect of the chemical composition on the catalytic performance of MgyAlOx catalysts for alcohol elimination reactions. J. Catal..

[68] MacKenzie K.J.D., Smith M.E. (2002). Multinuclear Solid-State Nuclear Magnetic Resonance of Inorganic Materials.

[69] Di Cosimo J.I., Díez V.K., Xu M., Iglesia E., Apesteguía C.R. (1998). Structure and surface and catalytic properties of Mg-Al basic oxides. J. Catal..

[70] Rebours B., d’Espinose de la Caillerie J.-B., Clause O. (1994). Decoration of nickel and magnesium oxide crystallites with spinel-type phases. J. Am. Chem. Soc..

[71] Bellotto M., Rebours B., Clause O., Lynch J., Bazin D., Elkaïm E. (1996). Hydrotalcite decomposition mechanism: A clue to the structure and reactivity of spinel-like mixed oxides. J. Phys. Chem..

[72] Reichle W.T., Kangand S.Y., Everhardt D.S. (1986). The nature of the thermal decomposition of a catalytically active anionic clay mineral. J. Catal..

[73] Rocha J., del Arco M., Rives V., Ulibarri M.A. (1999). Reconstruction of layered double hydroxides from calcined precursors: A powder XRD and ^27^Al MAS NMR study. J. Mater. Chem..

[74] McCarty R., Stebbins J.F. (2017). Constraints on aluminum and scandium substitution mechanisms in forsterite, periclase, and larnite: High-resolution NMR. Am. Mineral..

[75] Auerbach S.M., Carrado K.A., Dutta P.K. (2004). Handbook of Layered Material.

